# Skin-Related Adverse Reactions Induced by Oral Antidiabetic Drugs—A Review of Literature and Case Reports

**DOI:** 10.3390/ph17070847

**Published:** 2024-06-27

**Authors:** Justyna Kowalska, Dorota Wrześniok

**Affiliations:** Department of Pharmaceutical Chemistry, Faculty of Pharmaceutical Sciences in Sosnowiec, Medical University of Silesia in Katowice, Jagiellońska 4, 41-200 Sosnowiec, Poland; dwrzesniok@sum.edu.pl

**Keywords:** dipeptidylpeptidase-4 inhibitors, sulfonylureas, sodium-glucose cotransporter 2 inhibitors

## Abstract

Type 2 diabetes (T2DM) is a chronic metabolic disease with a steadily increasing prevalence worldwide. Diabetes affects the function of many organs, including the skin. Pharmacotherapy for T2DM is mainly based on oral hypoglycemic drugs. The therapeutic strategy is chosen taking into account the individual patient’s characteristics, among other comorbidities. Antidiabetic drugs can induce cutaneous adverse reactions (CADRs) ranging in severity from mild erythema to serious disorders such as DRESS or Stevens–Johnson syndrome. CADRs can result from hypersensitivity to the drug but can also be related to the mechanism of action of the drug or cross-reactivity with drugs of similar structure. This paper reviews CADRs induced by oral antidiabetic drugs, considering their dermatological manifestations and possible pathomechanisms. Particular attention was paid to specific dermatological conditions such as dipeptidylpeptidase 4 inhibitor-associated bullous pemphigoid or Fournier’s gangrene associated with sodium-glucose cotransporter 2 inhibitor therapy. Knowledge of the dermatological manifestations of CADRs is important in clinical practice. Recognition of a skin lesion resulting from an adverse drug reaction allows for appropriate management, which in this case is primarily related to drug discontinuation. This is particularly important in the treatment of T2DM since this disease has a high prevalence in the elderly, who are at higher risk of adverse drug reactions.

## 1. Introduction

Diabetes mellitus is a very serious health problem worldwide. The current global prevalence is estimated at 6.1%. The most prevalent diabetes type is type 2 (T2DM), which accounts for about 96% of the total incidence of diabetes [1,2]. T2DM was earlier known as insulin-independent diabetes mellitus or adult-onset diabetes because it most frequently occurs in people over the age of 45 and is characterized by a lack of insulin requirements to prevent ketoacidosis [3,4,5]. T2DM is caused by a combination of genetic factors and lifestyle. A major lifestyle risk factor is a high body mass index (BMI). Obesity has been found to account for about 55% of T2DM cases [2,6].

Pharmacotherapy for T2DM includes the use of oral and/or injectable hypoglycemic drugs. Patients are very often treated with two or three hypoglycemic drugs, as the combination of drugs with different mechanisms of action improves hyperglycemic control. Oral antidiabetic drugs include metformin, dipeptidylpeptidase-4 inhibitors (DPP-4-is), sodium-glucose cotransporter 2 inhibitors (SGLT-2is), sulfonylureas, meglitinides, thiazolidinediones, semaglutide (the only glucagon-like peptide 1 receptor agonist), alpha-glucosidase inhibitors (AGIs), and injectable drugs include glucagon-like peptide 1 receptor agonists and amylin mimetic [7,8].

According to the recommendations, the risk of adverse drug reactions, mainly hypoglycemia, volume depletion, and pancreatitis, among others, should be taken into account during the choice of antidiabetic drugs [9]. However, it should be noted that all drugs can induce hypersensitivity reactions, which manifest as skin lesions, among others. Oral antidiabetic drugs can cause skin reactions ranging from mild, such as erythema, to severe, such as toxic epidermal necrolysis [8,10]. Besides hypersensitivity reactions, skin lesions may result from the mechanism of action of the drug, cross-reactions, or the induction of other conditions that can lead to skin disorders. The current guidelines indicate that the new drugs that have been approved for therapy in recent years are first- and second-line drugs [9]. The safety of their use is still under evaluation, and post-marketing studies indicate a significantly higher risk of cutaneous side effects with some drug classes [11].

Diabetes is particularly common in people aged 65 and older in all countries, with a prevalence in this demographic group of over 20% worldwide [2]. In the elderly, polypharmacotherapy is often used due to the presence of comorbidities. In a high percentage of patients with T2DM, there is a need to use cardiological and antihyperlipidemic drugs. Elderly patients are at higher risk of adverse drug reactions. In addition, this group of patients has an increased risk of the prescribing cascade, which is the administration of drugs to treat the adverse reactions of drugs already in use [7].

It is important to know the extent of cutaneous adverse drug reactions (CADRs) to antidiabetic drugs because diabetes is prevalent in the elderly, who are at greater risk of side effects of therapy. It is also relevant because diabetic patients have a high risk of developing skin disorders due to the pathomechanism of the disease [12]. This review presents CADRs for oral antidiabetic drugs, taking into account available studies and case reports. We have presented a wide range of skin lesions, also taking into account conditions induced by oral antidiabetic drugs, which manifest as skin reactions, among other symptoms.

## 2. Pathophysiology of Skin-Related Disorders in Diabetes

The skin in diabetes undergoes pathological processes, whose severity is proportional to the duration of hyperglycemia [12]. It is estimated that 30% of diabetic patients have skin symptoms [10]. Cutaneous complications are associated with microangiopathy, neuropathy, and immune response changes, which are in turn due to biochemical alterations such as hyperglycemia, insulin deficiency, and non-enzymatic glycation (NEG) [10]. Insulin is essential for skin cell proliferation, differentiation, migration, metabolism, and apoptosis. Therefore, insulin deficiency leads to relevant skin abnormalities associated with, for example, impaired wound healing [13]. Enhanced NEG under hyperglycemic conditions decreases stiffness and reduces the elasticity of the skin due to collagen modification. In addition, NEG leads to an increase in oxidative stress and the induction of inflammation, both of which affect skin cell dysfunction [14]. Among others, suppression of proliferation and migration of keratinocytes and fibroblasts occurs [15,16]. Under hyperglycemic conditions, the physiological function of fibroblasts is impaired, leading to reduced secretion of extracellular matrix components. This results in decreased elasticity of the skin and causes it to become dry and thin [14,16]. Skin problems associated with T2DM include yellow nails, acrochordons, diabetic dermopathy, acanthosis nigricans, acquired perforating dermatosis, calciphylaxis, and eruptive xanthoma [17]. Skin complications of long-term diabetes also include infections, both bacterial and fungal [10,17,18].

## 3. Oral Antidiabetic Drugs

### 3.1. Metformin: Pharmacotherapy and Case Reports of Cutaneous Adverse Reactions

Metformin is a biguanide derivative that lowers blood glucose levels via multiple pathways. A drug reduces glucose absorption in the intestines and stomach and enhances insulin-dependent glucose uptake into skeletal muscle. In addition, it inhibits glycogenolysis and gluconeogenesis in the liver [19,20].

Metformin is a widely-used drug. It is recommended as a first-line treatment for T2DM according to the joint guidelines of the American Diabetes Association and the European Association for the Study of Diabetes [9]. In addition, metformin is recommended for the prevention of diabetes in patients aged < 60 years with a BMI ≥ 35 kg/m^2^ and women with a gestational diabetes history [21,22]. It is also used to reduce insulin resistance in patients with type 1 diabetes and overweight/obesity and to treat metabolic abnormalities associated with polycystic ovary syndrome [22,23].

This drug has a generally good safety profile and rarely causes CADRs. Metformin-induced skin lesions are immune-mediated reactions [24]. CADRs to metformin include, among others, erythema multiforme [25], lichen planus [26], rosacea-like facial rash [27], bullous pemphigoid [28], and psoriasiform drug eruption [29]. There are some reports in the literature of metformin-induced photosensitivity with the symptoms of erythematous, eczematous [30], or blistering lesions in sun-exposed areas [31]. Other skin adverse reactions caused by metformin are leukocytoclastic vasculitis and fixed-drug eruptions. Table 1 and Table 2 summarize the reported cases of these CADRs.

The most serious metformin-induced CADR that has been reported is a drug rash with eosinophilia and systemic symptoms syndrome known as DRESS syndrome. The patient presented with generalized pruritus, rash, lymphadenopathy, and eosinophilia [32].

Long-term metformin therapy can lead to the onset of vitamin B_12_ deficiency, which, besides hematological or neurological disorders, can manifest as hyperpigmented lesions on the knees, lateral surfaces of the legs, the dorsum of the hands and feet, fingers, as well as skin folds [33,34].

**Table 1:** Case reports of metformin-induced leukocytoclastic vasculitis.

Article	Patient Gender & Age	Latency Period	Level of Causility ^a^	Dermatologic Manifestation
Klapholz L. et al., 1986 [35]	F/59	4 months	NA	Purpuric eruption on forearms, buttocks, thighs, legs, and lower abdomen; arthralgia of ankles.
Salem Ch. et al., 2006 [36]	F/33	NA	Probable	Purpura on lower limbs.
Wiwanitkit V., 2011 [37]	F/35	NA	Probable	Purpura in the upper part of the lower limbs, near the genitals.
Czarnowicki T. et al., 2012 [38]	F/60	1 month	NA	Hemorrhagic papules, vesicles and bullae, located on the shins, thighs and buttocks.
Sheh T. and Tsai Ic., 2016 [39]	F/58	6 days	NA	Achy and tingling in legs, ulcerated sores on lower legs.
Vashisht T. et al., 2019 [40]	F/59	NA	NA	Purpuric skin lesions on arms, legs. and back.

NA  =  Not available; ^a^ The Naranjo Adverse Drug Reaction Probability Scale.

**Table 2:** Case reports of metformin-induced fixed-drug eruption.

Article	Patient Gender & Age	Latency Period	Level of Causility	Dermatologic Manifestation
Monroe J., 2010 [41]	F/41	NA	NA	Round, purplish-brown, targetoid macules on lips, face, and arms.
Steber C. et al., 2016 [42]	F/56	2 months	Definite ^a^/ Probable ^b^	Small, round, erythematic, slightly pustular lesions on palms and soles; painful erythematous skin around lesions on the soles; after rechallenge of metformin, lesions developed on the soles and on the dorsal side of the foot.
Amit S. et al., 2017 [43]	M/47	NA	Probable ^a^	Round/oval erythematous macules and palpable purpura patches on lower limbs, lower back, buttocks; pain while touching.
Ramírez-Bellver J. et al., 2017 [44]	M/86	NA	Probable ^a^	Round/oval, erythematous-violet, itchy macules and patches on the lower limbs, forearms and hands.
Togawa N. et al., 2019 [45]	M/46	10 days	NA	Skin rash on the left lower thigh and back that developed in the same areas after rechallenge of metformin.
Al Masri D. et al., 2021 [46]	F/58	2 weeks	NA	Itchy and burning blisters, ulcers and erythema on the right leg.
Abtahi-Naeini B. et al., 2023 [47]	M/43	5 years	Definite ^a^	Generalized bullous, erythematous to purple plaques located on the anterior and posterior trunk, upper and lower limbs, and mucous membranes of the genitals and lips; after rechallenge of metformin, bullous lesions with erosions and ulcers developed.

NA  =  Not available; ^a^ The Naranjo Adverse Drug Reaction Probability Scale; ^b^ The Kramer’s Scale.

#### Real-World Data on Metformin-Related Cutaneous Adverse Reactions

The FDA adverse reporting system (FAERS) lists 8712 cases of skin and subcutaneous tissue disorders related to metformin use. The largest number of cases included pruritus (1495), rash (1209), hyperhidrosis (1057), and urticaria (734) [48]. EudraVigilance notes 3624 cases of skin and subcutaneous tissue disorders [49].

### 3.2. Sulphonylureas: Pharmacotherapy and Case Reports of Cutaneous Adverse Reactions

Sulfonylureas are derivatives of sulfonamides, which are divided into two generations. The first-generation sulfonylureas, i.e., chlorpropamide and tolbutamide, are not currently used [50]. In contrast, the second-generation sulfonylureas, which include gliclazide, gliquidone, glipizide, glibenclamide (glyburide), and glimepiride, are among the most frequently used oral antidiabetic drugs [50]. This drug group is recommended when metformin is intolerant, contraindicated, or ineffective [51,52]. Sulfonylureas are also prescribed as an add-on to metformin to improve glycemic control [53]. 

The main mechanism of sulfonylureas is to trigger insulin release from the pancreatic β cells by binding to the sulfonylurea receptor. The drugs inhibit ATP-sensitive potassium channels, which leads to blockage of potassium ion efflux and depolarization of the cell membrane. As a result, the calcium channels open and the intracellular calcium concentration increases, which enhances insulin secretion [10,53,54,55].

CADRs occur in about 1% of patients using sulfonylureas [54]. First-generation drugs induced the following skin reactions: maculopapular exanthema, phototoxic and photoallergic reactions, pruritus, urticaria, erythema nodosum, fixed erythema, erythema multiforme, toxic epidermal necrolysis, and Lyell’s syndrome [10,56]. Moreover, chlorpropamide-alcohol flushing (CPAF) has been reported to occur in 10–30% of patients using the drug [10]. CPAF manifests as facial erythema after consuming a small amount of alcohol. It has been suggested that CPAF is an autosomal dominant inherited trait and that it has prognostic significance—patients with a positive CPAF test have a lower risk of developing vascular complications [57,58]. 

Second-generation sulfonylureas also induce skin reactions. In Table 3, case reports of CADRs induced by these drugs are summarized. The Naranjo probability scale was applied in the studies by Ben Salem et al. [59] and Al-Badawi et al. [60] to verify whether there was a causal relationship between the observed CADR and the sulfonylurea used. In both cases, a probable association was found. Ozuguz et al. [61] obtained a positive patch test result for gliclazide. In in vitro and in vivo studies, the phototoxic potential of the following second-generation sulfonylureas has been demonstrated: glipizide, glibenclamide, and gliquiudone [62,63]. 

CADRs to sulfonylureas may result from cross-reactivity with other sulfonamide derivatives. Sulfonamides are associated with a high risk of allergic reactions. Sulfonamide metabolites act as haptens and bind to proteins, and the formed complexes, after being bound to MHC class I or II molecules, are presented to T lymphocytes. Sulfonylureas can trigger an allergic response similar to sulfonamides due to their structural similarity [64,65]. The following cases of cross-reactions between sulfonamide derivatives have been reported:

- Petechial eruptions after the use of hydrochlorothiazide in a patient diagnosed with tolbutamide allergy [66];
- Leukocytoclastic vasculitis after gliburide use in a patient with a history of sulfamethoxazole-trimethoprim allergy [67];
- Erythema multiforme occurring after the use of celecoxib as well as glyburide [68];
- Fatal toxic epidermal necrolysis probably associated with glimepiride in a patient with sulfamethoxazole-trimethoprim hypersensitivity [69].

**Table 3:** Case reports of cutaneous adverse reactions induced by second-generation sulfonylureas.

Article	Drug	Patient Gender & Age	Latency Period	Type of Cutaneous Adverse Reaction	Dermatologic Manifestation
Adams B.B. et al., 1999 [70]	Glipizide	M/66	2 weeks	Pigmented purpuric dermatosis	Multiple, scattered, scaly, erythematous to light brown macules on the upper legs and buttocks.
Noakes R., 2003 [71]	Glimepiride	M/63	3 months	Lichenoid drug eruption	Non-itchy, purple papules on both lower extremities.
Duncan C. et al., 2004 [72]	Glibenclamide	M/67	4 weeks	Stevens–Johnson syndrome	Erythema of face and neck, peri-orbital oedema with mucocutaneous ulceration of the mouth, extensive maculopapular eruption on the limbs.
Ben Salem C. et al., 2007 [59]	Glimepiride	F/72	6 weeks	Leukocytoclastic vasculitis	Erythematous papules on lower and upper limbs, and the trunk.
Contreras-Steyls M. et al., 2013 [73]	Gliclazide	F/78	3 days	Acute generalized exanthematous pustulosis	Erythematous, slightly itchy eruption on the face, upper limbs, trunk and thighs.
Henley J.K. et al., 2013 [74]	Glyburide	M/71	2 weeks	Leukocytoclastic vasculitis	Palpable purpura on the lower extremities.
Ozuguz P. et al., 2014 [61]	Gliclazide	F/70	3 days	Erythroderma	Erythema and desquamation covering almost the entire body surface.
Chadli Z. et al., 2018 [75]	Glibenclamide	F/75	NA	Photodistributed lichenoid eruption	Skin eruption on the face, neck and hands.
Dev N., 2018 [76]	Glimepiride	M/40	1 month	Drug reaction with eosinophilia and systemic symptoms	Erythematous rash along with scaling on the face, trunk and extremities; swelling over the lower limbs; yellowish discoloration of eyes.
Ajaaouani R. et al., 2022 [77]	Gliclazide	F/68	3 months	Lichen planus pemphigoid	Generalized polygonal and purple papules ending in tense blisters on the abdomen, trunk, back, legs, and thighs.
Al-Badawi S. et al., 2023 [60]	Gliclazide	F/72	NA	Erythroderma	Skin rash on abdomen, lower limbs, arms, and back; the rash evolved from small spots into larger, painful, itchy lesions.

NA  =  Not available.

#### Real-World Data on Sulphonylureas-Related Cutaneous Adverse Reactions

The FAERS reports 4073 cases of skin and subcutaneous tissue disorders associated with the use of second-generation sulfonylureas: 24 for gliquidone, 816 for gliclazide, 929 for glimepiride, 995 for glipizide, and 1309 for glyburide (glibenclamide). The most commonly reported CADRs include rash, pruritus, and dermatitis [48]. The EudraVigilance lists 1923 cases of skin and subcutaneous tissue disorders: 21 for gliquidone, 162 for glipizide, 380 for glyburide (glibenclamide), 659 for gliclazide, and 701 for glimepiride [49].

### 3.3. Sodium-Glucose Cotransporter 2 Inhibitors: Pharmacotherapy and Case Reports of Cutaneous Adverse Reactions

Sodium-glucose cotransporter 2 inhibitors are recommended as second-line treatment after metformin for T2DM patients with atherosclerotic cardiovascular disease, heart failure, or chronic kidney disease [9]. SGLT-2is include canagliflozin, dapagliflozin, empagliflozin, ertugliflozin, bexaglifloxin, ipragliflozin, and sotagliflozin [78]. These drugs act by inhibiting paired sodium and glucose reuptake in the renal proximal tubule and thus supporting urinary excretion of glucose and sodium [78,79].

CADRs usually appear within 2 weeks of SGLT-2is administration and include, among others, severe generalized rash, urticaria, erythema, drug eruption, and eczema [80,81]. In addition, a case of bullous pemphigoid during treatment with ipragliflozin [82], generalized intense pruritus caused by canagliflozin [83], and a fixed drug eruption induced by dapagliflozin [84] were reported. In reports by Yabe et al. [81] and Sakaeda et al. [85], ipraglifrozin, within SGLT-2is, was associated with the highest incidence of skin and subcutaneous tissue disorders. A significant risk of ipraglifrozin-induced CADRs may result from the interaction of the drug with melanin, a pigment occurring in the skin. A 3-D in silico docking simulation showed that ipragliflozin can bind to melanin, and analysis of the drug’s skin tissue distribution in rats showed that ipragliflozin was retained in skin tissue [85]. Studies by Yokote et al. [86] and Maegawa et al. [87] found that female patients and those older than age 65 have a higher risk of skin complications during ipragliflozin treatment.

Ma et al. [88] showed that SGLT-2 significantly increased the risk of psoriasis in patients with diabetes and renal diseases. 

SGLT-2 increases the risk of urinary tract infections due to their mechanism of action. The drugs induce glucosuria, and increased glucose concentration in the urine creates a rich medium for bacteria and fungi [80,89]. It is estimated that 5–10% of women with T2DM receiving SGLT-2is experience genital fungal infections. Many genitourinary infections are mild to moderate and are readily treated. However, there is a risk of developing severe urosepsis or potentially life-threatening Fournier’s gangrene [11,89]. Fournier’s gangrene is necrotizing fasciitis of the perineum. Clinical manifestations include persistent severe perianal or genital pain, blistering, redness, ecchymosis, skin necrosis, and extensive swelling [90,91]. It has not yet been determined whether SGLT-2 itself causes Fournier’s gangrene or whether a subgroup of patients receiving those antidiabetic drugs are at risk. The following conditions are considered to be risk factors: advanced age, obesity, smoking, and alcohol abuse [78,92,93]. Table 4 summarizes cases of Fournier’s gangrene in patients using SGLT-2is. In a study by Nagano et al. [91], the Naranjo probability scale indicated a probable relationship between Fornier’s gangrene and empagliflozin.

#### Real-World Data on Sodium-Glucose Cotransporter 2 Inhibitors-Related Cutaneous Adverse Reactions

The disproportionality analysis conducted by Raschi et al. [11] showed a higher frequency of reported skin-related side effects associated with SGLT-2is administration, which was unexpected as it did not emerge from preapproval randomized clinical trials. The FAERS notes 6123 cases of skin and subcutaneous tissue disorders linked to the use of SGLT-2is: 1 for bexaglifloxin, 2 for ipragliflozin, 4 for sotagliflozin, 1203 for dapagliflozin, 1956 for empagliflozin, and 2957 for canagliflozin. Among the most commonly reported CADRs are: diabetic foot, skin ulcer, rash, pruritus, and angioedema [48]. The EudraVigilance holds 5638 records of skin and subcutaneous tissue disorders: 19 for ertugliflozin, 1677 for dapagliflozin, 1884 for empagliflozin, and 2058 for canagliflozin [49].

### 3.4. Dipeptidylpeptidase-4 Inhibitors: Pharmacotherapy and Case Reports of Cutaneous Adverse Reactions

Dipeptidylpeptidase-4 inhibitors, also known as gliptins, are a class of oral antidiabetic drugs that include alogliptin, anagliptin, linagliptin, saxagliptin, sitagliptin, teneligliptin, and vildagliptin. Gliptins are recommended as second-line treatment when metformin is contraindicated or intolerant and in patients without high-risk or diagnosed cardiovascular disease (as SGLT-2is are preferred in this patient subgroup) [104,105,106]. Gliptins, by inhibiting the enzyme dipeptidylpeptidase-4, prevent the degradation of the incretin hormone (glucagon-like peptide 1), which has a role in controlling post-meal glycemic levels. Incretin promotes insulin secretion from β cells in a glucose-dependent manner and suppresses glucagon release from α cells [107,108,109].

According to a report by Huang et al. [110], gliptins among non-insulin antidiabetic drugs are associated with a higher number of reported skin-related adverse reactions. Prescribing information in the US and European Union for alogliptin, sitagliptin, saxagliptin, and vildagliptin (European Union only) contains warnings and precautions about hypersensitivity reactions affecting the skin [111]. The hypersensitivity to gliptins can manifest as angioedema, oedema peripheral, pruritus, rash, rash generalized, urticaria, or pemphigoid [110]. There have been reports of linagliptin-related blistering and ulceration [112] and vildagliptin-related spongiotic dermatitis [113]. Whereas, the following skin reactions were observed in sitagliptin-treated patients: maculopapular-type eruption [114], photosensitivity [115], bullous lichenoid dermatitis [113], pemphigus vulgaris [116], as well as Stevens–Johnson syndrome [117]. Other reported CADRs during gliptin therapy include lichenoid dermatitis and psoriasiform dermatitis [113,118].

One of the notable skin disorders induced by DPP-4-is is bullous pemphigoid, named as dipeptidylpeptidase 4 inhibitor-associated bullous pemphigoid (DPP4i-BP) or gliptin-associated bullous pemphigoid (GABP) [119,120]. Table 5 presents GABP case reports, including clinical manifestations. 

It has been reported that vildagliptin is the DPP-4-i that most frequently induces the onset of BP [121,122,123,124,125]. A higher risk of GABP has been shown to occur in elderly patients and those with dementia [126,127]. The latency between the beginning of gliptin treatment and the onset of BP symptoms varied widely across studies and ranged from 8 days to 6.5 years [128]. Several Asian studies have shown that patients with GABP exhibit a non-inflammatory phenotype characterized by less eosinophilia infiltration than observed in typical BP [129,130,131]. In contrast, European studies did not confirm significant differences between GABP and typical BP [122,132,133].

The pathomechanism of GABP remains largely unclear. It seems significant that DPP-4 is involved in many biological processes, is expressed as a cell surface protein by various cells, including T lymphocytes, macrophages, fibroblasts, and keratinocytes, and is also found in body fluids [113,134]. Among others, DPP-4 is a cell-surface plasminogen receptor that is responsible for the transformation of plasminogen into plasmin. Plasmin cleaves collagen XVII, which plays a crucial role in the maintenance of stable adhesion of the epidermis to the dermis. It is supposed that DPP-4 suppression modifies the ability of plasmin to cleave collagen XVII and could affect the formation of new epitopes for BP autoantibodies. In addition, DPP-4 inhibition is also thought to increase the activity of proinflammatory chemokines such as CCL11/eotaxin, enhancing the activation of eosinophils in the skin and bullae formation [119,128,135].

**Table 5:** Case reports of dipeptidylpeptidase-4 inhibitor-associated bullous pemphigoid.

Article	Drug	Patient Gender & Age	Latency Period	Level of Causility	Dermatologic Manifestation
Pasmatzi E. et al., 2011 [136]	Vildagliptin	F/59	2 months	NA	Itchy blistering eruptions
	Vildagliptin	M/67	2 months	NA	Pruritic bullous lesions
Attaway A. et al., 2014 [137]	Sitagliptin	M/70	1 year	Possible ^a^	Bullae eruptions on the neck, chest, arms, and groin
Béné J. et al., 2015 [138]	Vildagliptin	F/86	2 months	NA	Erythematous bullous eruption; extensive erosions
	Vildagliptin	M/79	37 months	NA	Erythematous vesicular bullous eruption on the trunk and the upper and lower limbs
	Vildagliptin	F/77	26 months	NA	Extensive pruriginous bullous eruption
García M. et al., 2016 [139]	Vildagliptin	F/74	1 year	Probable ^b^	Itchy, vesicular skin lesions on the trunk and a two-centimeter erythematous plaque on the left arm with peripheral bullae
Haber R. et al., 2016 [140]	Linagliptin	M/60	4 months	NA	Itchy, erythematous, tense bullae on the extremities
	Linagliptin	F/70	3 months	NA	Itchy and tense bullae on the trunk
Keseroglu H.O. et al., 2016 [141]	Vildagliptin	F/61	1 year	NA	Tense bullae on the arm and crusted erythematous eruptions on the chest, upper extremities, back, and hips.
Mendonça F.M. et al., 2016 [142]	Linagliptin	M/82	45 days	Probable/ Likely ^c^	Itchy cutaneous eruption characterized by small bullae.
	Vildagliptin	F/72	3 months	Probable ^c^	Itchy, tense bullae on urticarial base
Esposito I. et al., 2017 [143]	Linagliptin	F/73	5 months	Probable ^a^	Numerous pruritic, tense blisters on an erythematous base and multiple erosions on the face, trunk, and extremities.
Harada M. et al., 2017 [144]	Sitagliptin	M/78	3 years	NA	Skin bullae on both extremities.
Oya K. et al., 2017 [145]	Anagliptin	M/84	1 month	NA	Erythematous, itchy, blistering eruption and erosion on the trunk.
Sakai A. et al., 2017 [146]	Linagliptin	F/76	9 months	NA	Great number of erosions and tense blisters spread over the whole body.
Mai Y. et al., 2018 [147]	Linagliptin	M/60	1 year	NA	Bullae and erosions over the entire body.
Maki N. et al., 2018 [148]	Teneligliptin	M/70	1 month	NA	Multiple tense bullae and scabby papules and nodules on the trunk.
Yoshiji S. et al., 2018 [149]	Anagliptin	M/63	5 months	Reasonable ^c^	Erythematous, bullous eruptions over the whole body.
	Linagliptin	M/81	8 months	Reasonable ^c^	Erythematous, tense bullae that first appeared on the thigh and spread over the body.
	Linagliptin	F/86	9 months	Reasonable ^c^	Erythematous, tense bullae on the back that spread over the body.
	Linagliptin	F/83	10 months	Reasonable ^c^	Erythematous tense bullae.
	Vildagliptin	F/86	6 months	Reasonable ^c^	Erythematous tense bullae.
Karthik S. et al., 2019 [150]	Vildagliptin	M/61	6 months	NA	Painless, itchy bullae on the extremities spreading to the body and arms.
Hibi A. et al., 2020 [151]	Linagliptin	M/56	5 months	NA	Tense bullae spread over the entire body.
Jha A. et al., 2020 [152]	Linagliptin	M/52	4 months	Probable ^c^	Tense, itchy bullous lesions on the soles, abdomen, and upper extremities.
	Linagliptin	F/70	7 months	Probable ^c^	Tense bullous lesions on the trunk and extremities.
	Linagliptin	M/42	2 months	Probable ^c^	Numerous small vesicular lesions on the right upper limb with intense pruritus and erythema.
	Linagliptin	F/76	5 months	Probable ^c^	Small, itchy, bullous lesions on lower extremities and abdomen.
	Linagliptin	F/67	20 days	Probable ^c^	Bullous itchy lesions on the upper back.
	Linagliptin	M/63	21 days	Probable ^c^	Numerous itchy bullous lesions on both upper limbs.
	Linagliptin	F/64	20 days	Probable ^c^	Small, fluid-filled vesicular lesions on the left cheek.
	Linagliptin	M/56	14 days	Certain ^c^	Numerous tense bullous lesions on the trunk, oral mucosa, and extremities.
	Sitagliptin	M/80	3 months	Certain ^c^	Numerous itchy blistering lesions with erythema on the upper extremities.
	Vildagliptin	F/56	9 months	Probable ^c^	Itchy bullous lesions on the soles and abdomen.
	Vildagliptin	M/61	1 year	Probable ^c^	Bullous lesions on the abdomen and dorsum of the hands.
	Vildagliptin	M/52	2 months	Probable ^c^	Bullous itchy lesions on the chest and right arm.
	Vildagliptin	F/63	2 years	Probable ^c^	Maculopapular, intensely pruritic lesions on both upper extremities and breasts; small bullous lesions on the trunk.
Dbouk S. et al., 2021 [153]	Linagliptin	F/77	2 years	NA	Numerous erythematous papules and nodules on the upper limbs and trunk.
Kitaoa R. et al., 2021 [154]	Teneligliptin	M/60	41 months	NA	Tense bullae that appeared on the legs and spread to the trunk and arms.
Duraisamy P. et al., 2022 [113]	Linagliptin	M/67	12 months	Possible ^a^	Bullae with surrounding erythema on the legs.
	Saxagliptin	M/49	10 months	Possible ^a^	Hyperpigmented plaques and macules on the chest; bullae on the legs.
	Sitagliptin	F/73	6 months	Possible ^a^	Numerous bullae on the legs.
	Sitagliptin	M/89	4 months	Possible ^a^	Numerous bullae on the legs and erosions on the thighs and groins.
	Teneligliptin	F/88	18 months	Possible ^a^	Numerous erosions, bullae, and edematous plaques on the abdomen, trunk, and thighs.
	Teneligliptin	M/81	2 months	Possible ^a^	Numerous bullae on lower legs and thighs.
	Teneligliptin	F/59	18 months	Possible ^a^	Few bullae on the legs and feet along with surrounding erythema and oedema.
	Teneligliptin	F/72	1 month	Possible ^a^	Bullae with surrounding erythema on the neck, chest, and arms.
	Teneligliptin	M/68	6 months	Possible ^a^	Few bullae on the legs and back.
	Vildagliptin	M/65	6 months	Possible ^a^	Tense bullae with erythema on the abdomen, back, thighs, and legs.
	Vildagliptin	F/48	6 months	Possible ^a^	Bullae on forearms; erosions on buttocks and back.
	Vildagliptin	M/69	12 months	Possible ^a^	Numerous bullae with light erythema and erosions on the neck, chest, forearms, and legs.
Charitaki E. et al., 2023 [155]	Linagliptin	F/75	35 months	Probable ^a^	Eczema-like lesions in the upper back.
	Linagliptin	M/82	29 months	Probable ^a^	Numerous bullae and itching on both legs.
	Vildagliptin	M/85	23 months	Probable ^a^	Widespread, erythematous rash with bullae, blisters, and erosions affecting the arms, legs, front of the chest, abdomen, and back.
	Vildagliptin	M/65	9 months	Probable ^a^	Large tense bullae on the trunk and thighs.
	Vildagliptin	M/71	10 months	Probable ^a^	Tense vesicles and bullae on an erythematous base on the back and arms.

NA  =  Not available; ^a^ The Naranjo Adverse Drug Reaction Probability Scale; ^b^ The Karch and Lasagna Scale; ^c^ The WHO-UMC System for Standardised Case Causality Assessment.

#### Real-World Data on Dipeptidylpeptidase-4 Inhibitors-Related Cutaneous Adverse Reactions

The FAERS reports 3024 cases of skin and subcutaneous tissue disorders arising after DPP-4-is use: eight for anagliptin, forty-one for teneligliptin, sixty-six for saxagliptin, ninety-seven for alogliptin, three hundred and two for sitagliptin, seven hundred and three for vildagliptin, and one thousand seven hundred and seven for linagliptin. The most commonly reported CADR is pemphigoid—970 cases [48]. The EudraVigilance lists 6419 cases of skin and subcutaneous tissue disorders: 283 for saxagliptin, 342 for alogliptin, 1603 for linagliptin, 1720 for vildagliptin, and 2471 for sitagliptin [49].

The relationship between gliptins and BP, as well as the clinicopathological and immunological features of GABP, have been widely studied. The following reports confirm the increased risk of BP in gliptin-treated patients:

- A Swiss case-controlled study by Schaffer et al. [156];
- A French/Swiss case-controlled study by Benzaquen et al. [121];
- A French case-controlled study by Plaquevent et al. [122];
- An Israeli case-controlled study by Kridin and Bergman [123];
- A Korean case-controlled study by Lee et al. [124];
- A Greek prospective observational study by Lambadiari et al. [157];
- A case–noncase study in the French Pharmacovigilance Database by Béné et al. [158];
- A Finnish nationwide Registry study by Varpuluoma et al. [125];
- A large population-based study in the UK by Douros et al. [159];
- A meta-analysis by Phan et al. [160];
- A meta-analysis by Yang et al. [161].

### 3.5. Semaglutide: Pharmacotherapy and Case Reports of Cutaneous Adverse Reactions

Semaglutide is the first oral glucagon-like peptide 1 receptor agonist approved for the treatment of T2DM. It is recommended as a first- or second-line therapy [162]. Semaglutide, as administered subcutaneously, is also approved for the treatment of overweight and obesity [163]. A case of bullous pemphigoid associated with semaglutide was reported, which manifested as crusted erosions of the breast and lower back after 1 month of therapy [164]. In addition, there is a case report of itchy palmar erythema 17 h after the administration of the drug [165].

#### Real-World Data on Semaglutide-Related Cutaneous Adverse Reactions

In the case of sameglutide, 2513 cases of adverse skin and subcutaneous tissue disorders were reported in the FAERS and 1425 in the EudraVigilance [48,49]. The following CADRs had the largest number of reports: rash, pruritus, alopecia, and hyperhidrosis [48].

### 3.6. Thiazolidinediones: Pharmacotherapy and Case Reports of Cutaneous Adverse Reactions

Thiazolidinediones decrease insulin resistance by activating the gamma isoform of the peroxisome proliferator-activated receptor, which regulates the transcription of genes involved in glucose and lipid metabolism. Thiazolidinediones, which include pioglitazone and rosiglitazone, are recommended as third-line or second-line drugs if first- and second-line antidiabetic drugs are not effective [166,167]. CADRs in thiazolidinedione therapy are primarily related to hypersensitivity reactions. A case report of hypersensitivity to pioglitazone has been reported, which manifested as pruritus and rash and appeared after 2 months of therapy [168].

#### Real-World Data onon Thiazolidinediones-Related Cutaneous Adverse Reactions

The FAERS reporting system records 183, and the EudraVigilance dtabase records 679 cases of skin and subcutaneous tissue disorders in response to pioglitazone (respectively 145 and 38) and rosiglitazone (respectively 393 and 286) [48,49].

### 3.7. Meglitinides: Pharmacotherapy and Case Reports of Cutaneous Adverse Reactions

Meglitinides are a group of oral antidiabetic drugs preferred in patients with chronic or end-stage renal disease. They stimulate the production of endogenous insulin in the pancreas. The Meglitinides group comprises repaglinide, mitiglinide, and nateglinide [169,170]. Reported CADRs to the meglitinide therapy include rash, pruritus, and pemphigoid [24]. A repaglinide hypersensitivity case report is available in the literature [171]. The clinical manifestation was a maculopapular rash on the face, neck, and upper chest, which appeared after 5 days of therapy.

#### Real-World Data onon Meglitinides-Related Cutaneous Adverse Reactions

The FAERS notes 634 cases of skin and subcutaneous tissue disorders linked to the use of meglitinides: eight for mitiglinide, one hundred and twenty-four for nateglinide, and five hundred and two for repaglinide [48]. The EudraVigilance lists four hundred and twenty-five cases of skin and subcutaneous tissue disorders: fourteen for mitiglinide, eighty-four for nateglinide, and three hundred and twenty-seven for repaglinide [49].

### 3.8. Alpha-Glucosidase Inhibitors: Pharmacotherapy and Case Reports of Cutaneous Adverse Reactions

Alpha-glucosidase inhibitors are used in the treatment of T2DM and glucose intolerance. They are particularly useful for patients who cannot use sulfonylurea derivatives and metformin because of the risk of hypoglycemia or lactate acidosis. AGIs include acarbose, voglibose, and miglitol. AGIs inhibit the absorption of carbohydrates from the small intestine. They competitively inhibit enzymes, including alpha-glucosidase, which is responsible for converting complex, non-absorbable carbohydrates into simple, absorbable carbohydrates [169,172].

The most commonly used drug among the AGIs is acarbose [172]. Acarbose is poorly absorbed from the gut and has low bioavailability, so it rarely causes adverse reactions [169,173]. Among CADRs, a case of acarbose-induced generalized erythema multiforme was reported [173]. The patient presented with erythematous plaques with vesicles on almost the entire body after 13 days of therapy.

#### Real-World Data onon Alpha-Glucosidase Inhibitors-Related Cutaneous Adverse Reactions

The FAERS reports 541 cases of skin and subcutaneous tissue disorders arising after AGIs use: 71 for voglibose, 74 for miglitol, and 396 for acarbose. Among the most commonly reported CADRs are hyperhidrosis and pruritus. Voglibose has had 11 cases of Stevens–Johnson syndrome 48. The EudraVigilance lists 508 cases of skin and subcutaneous tissue disorders: 57 for voglibose, 58 for miglitol, and 393 for acarbose49.

In addition, a population-based study by Huang et al. [174] found that AGIs use and discontinuation may be associated with an increased risk of psoriatic disease. The data source was the 1999–2013 Taiwanese Longitudinal Cohort of Diabetes Patients Database. It was considered the data of patients who used the following AGIs: acarbose and miglitol.

## 4. Methods

This review compiles case reports following electronic searches conducted between October and December 2023 in the PubMed and Google Scholar databases. No time limitations were imposed. The following queries were used for Pubmed: (i) (metformin) AND (skin); (ii) (chlorpropamide OR tolbutamide OR gliclazide OR gliquidone OR glipizide OR glibenclamide OR glyburide OR glimepiride) AND (skin); (iii) (canagliflozin OR dapagliflozin OR empagliflozin OR ertugliflozin OR bexaglifloxin OR ipragliflozin OR sotagliflozin) AND (skin); (iv) (alogliptin OR anagliptin OR linagliptin OR saxagliptin OR sitagliptin OR teneligliptin OR vildagliptin) AND (skin); (v) (semaglutide) AND (skin); (vi) (pioglitazone OR rosiglitazone) AND (skin); (vii) (repaglinide OR mitiglinide OR nateglinide) AND (skin); (viii) (acarbose OR voglibose OR miglitol) AND (skin); Searching Google, the query paired the word “skin” with the name of the drug. In particular, articles readily available in English, both original and translated, were included, and others were excluded. We retained articles that met the following criteria: (1) The authors suggested the possibility of an association between the induction of a skin-related effect and the use of the antidiabetic drug; (2) the clinical symptoms observed in the patient were presented. We discarded papers that were not case reports or case series.

Data from the FAERS and EudraVigilance databases indicate the total number of available reports while searching these databases for the preparation of the review (May 2024).

In the case reports shown, we presented the results of the assessment of the causality of adverse drug reactions, taking into account the following methods [175,176,177]:

- The Naranjo Adverse Drug Reaction Probability Scale—a questionnaire consisting of 10 questions with “yes”, “no”, and “unknown” answers—is used. The score is the sum of the values assigned to each item. Based on the score, the causality is assessed as definite, probable, possible, or doubtful.
- The Karch and Lasagna Scale—it consists of three tables with a number of closed questions, which should be answered dichotomously. Causality is classified as definite, probable, possible, conditional, or unlikely.
- The Kramer’s Scale—it consists of 56 questions and is based on a comprehensive evaluation of all evidence available, including the patient’s history and laboratory tests. It classifies causality as certain, probable, possible, or unlikely.
- The WHO-UMC System for Standardised Case Causality Assessment—it is categorized into six groups based on four criteria: temporal relationship, absence of other competing causes, laboratory findings, and de-challenge and re-challenge. Causality is classified as certain, probable/likely, possible, unlikely, conditional/unclassified, or unassessable/unclassifiable.

## 5. Conclusions

This review shows that oral hypoglycemic agents that are first- or second-line drugs cause various CADRs. Among others, the widely used sulfonylureas may cause skin lesions due to cross-reactions with other sulfonamide derivatives used, e.g., in the treatment of hypertension. The use of new antidiabetic drugs approved for treatment in recent years, such as dipeptidylpeptidase-4 inhibitors and sodium-glucose cotransporter 2 inhibitors, is associated with an increased risk of dermatological conditions related to the mechanisms of action of these drugs. Inhibition of dipeptidylpeptidase-4 activity may lead to the onset of bullous pemphigoid, and induction of glucosuria by sodium-glucose cotransporter 2 inhibitors may promote the development of Fournier’s gangrene. 

Management of adverse drug reactions primarily involves discontinuing the drug. Therefore, it is important to be aware of the clinical manifestations of CADRs induced by oral antidiabetic drugs to properly diagnose the skin lesion and avoid the use of additional drugs. This is significant in medical practice, as diabetes occurs mainly in the elderly, who show a high risk of a prescribing cascade.

## Figures and Tables

**Table 4:** Case reports of sodium-glucose cotransporter 2 inhibitors-induced Fournier’s gangrene.

Article	Drug	Patient Gender & Age	Latency Period
Chi W.C. and Lim-Tio S., 2016 [94]	Dapagliflozin	M/67	3 weeks
Kumar S. et al., 2017 [90]	Empagliflozin	M/41	7 months
Omer T. et al., 2018 [95]	Dapagliflozin	M/60	5 months
Elshimy G. et al., 2019 [96]	Empagliflozin	M/57	10 days
Nagano Y. et al., 2019 [91]	Empagliflozin	M/34	142 days
Onder C.E. et al., 2019 [97]	Dapagliflozin	M/64	6 months
Rodler S. et al., 2019 [98]	Dapagliflozin	M/39	4 years
Elbeddini A. et al., 2020 [99]	Dapagliflozin	F/71	5 years
Ellegård L. and Prytz M., 2020 [100]	Dapagliflozin	F/52	1.5 years
Garcıa-Garcıa A. et al., 2020 [101]	Dapagliflozin	M/68	NA
Kasbawala K. et al., 2020 [102]	Canagliflozin	F/37	1 month
Moon J.Y. et al., 2021 [103]	Dapagliflozin	M/66	NA

NA  =  Not available.

## Data Availability

Data sharing is not applicable.
